# Astragaloside IV Alleviates Ulcerative Colitis Progression by Inhibiting WDR5‐Mediated ENO1 H3K4me3 Modification

**DOI:** 10.1002/kjm2.70064

**Published:** 2025-06-30

**Authors:** Su‐Xiao Wu, Zi‐Lan Chen, Xiao‐Hong Wang, Jie Gu

**Affiliations:** ^1^ Department of Gastroenterology Suzhou Hospital of Integrated Traditional Chinese and Western Medicine Suzhou Jiangsu People's Republic of China

**Keywords:** Astragaloside IV, ENO1, H3K4me3, ulcerative colitis, WDR5

## Abstract

Ulcerative colitis (UC) has become a prevalent global health concern. This study scrutinized the influence of Astragaloside IV (ASI) on DSS‐induced UC, with particular emphasis on the role of WDR5 in mediating ENO1 expression. The therapeutic efficacy of ASI was assessed in a mouse model of UC by evaluating disease activity index, pathology, colon length, and inflammatory factor contents. Through bioinformatics analysis, the UC‐related differentially expressed genes were predicted using the GSE38713 database and intersected with the lists of ASI targets and transcription factors, and the protein–protein network was constructed to screen the key target transcription factors. ASI inhibited the shortening of colon length, reduced histological damage scores, ameliorated pathology, and the overproduction of pro‐inflammatory cytokines. After ASI treatment, DSS‐stimulated human NCM460 cells showed increased cell viability, decreased levels of pro‐inflammatory cytokines and cleaved‐Caspase‐3, and enhanced ZO‐1 and claudin‐3 expression. WDR5 was a target of ASI in UC, and overexpression of WDR5 compromised the effects of ASI. WDR5 promoted the H3K4me3 modification of the ENO1 promoter and thereby regulated ENO1 transcriptional activation. Silencing of ENO1, again, repressed NCM460 cell apoptosis and alleviated UC‐like symptoms in mice. In conclusion, ASI mitigated UC by inhibiting WDR5 and reducing H3K4me3‐mediated ENO1 activation.

## 1 Introduction

Ulcerative colitis (UC) is a chronic inflammatory disease of the intestines characterized by persistent or recurrent mucous pus, bloody stool, diarrhea, abdominal pain, and different degrees of systemic symptoms, principally disturbing young to middle‐aged individuals [1]. While medical therapy aims to induce remission, optimal management of mild to moderate UC is still challenging because of heterogeneity in severity classifications and non‐standardized approaches [2]. Mesalazine, as a first‐line treatment for inflammatory bowel disease, might result in a series of side effects, including gastrointestinal issues (8.2% or 11.9% depending on the dose), headache (about 11%), hypersensitivity or UC exacerbation (0.5%–6.5%), as well as pulmonary adverse events (nasopharyngitis as the most common one) [3]. Chinese medicine, on the other hand, has seen increasing value in treating UC [4, 5].

*Astragalus membranaceus* is a traditional Chinese medicine, and its constituents have been reported to exhibit multiple bioactivities, such as anti‐inflammatory, antioxidant, immune regulatory, hypolipidemic, hypoglycemic, hepatoprotective, and diuretic properties [6]. More recently, Astragaloside IV (ASI), a terpenoid extracted from *Astragalus membranaceus*, has been reported to reduce various types of inflammatory injuries, such as ischemia–reperfusion injury, allergic diseases, diabetes mellitus and its complications, and neurological disorders [7, 8, 9]. More relevantly, it has shown attenuating effects on UC by improving the intestinal epithelial barrier [10]. However, the molecular mechanism involved remains to be further explored. The intestinal physical barrier primarily consists of intestinal epithelial cells, tight junctions, and extracellular mucus, and tight junctions, including transmembrane proteins like claudin, occludin, and Zonula occludens protein 1 (ZO‐1), connect adjacent intestinal epithelial cells, forming the physical barrier between the apical and the basolateral plasma membrane domains [11]. Therefore, targeting the intestinal mucosal barrier might be an effective way to treat UC.

WD repeat‐containing protein 5 (WDR5), belonging to the WD40 protein family, is widely involved in various biological activities and recognizes the methylated fourth lysine of histone H3, histone 3 lysine 4 (H3K4) [12]. The H3K4me3 marker acts as an activator of gene expression [13]. Interestingly, long noncoding (lnc) RNA PTPRE‐AS1 bound WDR5 directly, thereby modulating H3K4me3 of the PTPRE promoter to modulate PTPRE‐dependent signaling during M2 macrophage activation, which is involved in inflammatory diseases, including reducing chemical‐induced colitis [14]. However, the sole role of WDR5 in UC and whether its expression was controlled by ASI has not been addressed. In this study, a dextran‐sulphate‐sodium (DSS)‐induced mouse model [15] and human‐derived colonic epithelial NCM460 cells were established to explore the potential protective effects of ASI against UC and further elucidate the potential mechanism by WDR5‐mediated H3K4me3 modification.

## 2 Materials and Methods

### 2.1 Establishment of DSS‐Induced UC Model in Mice

Female C57BL/6 mice (20 ± 2 g, 8 weeks old) were purchased from Aniphe Biolaboratory Inc. (Nanjing, Jiangsu, China) and housed in the animal facilities with a 12:12‐h light/dark cycle, controlled temperature (26°C ± 2°C), and humidity (55% ± 10%), for a 3‐days quarantine. All procedures were approved by the Animal Care and Use Committee of Suzhou Hospital of Integrated Traditional Chinese and Western Medicine and conducted in adherence to the Guide for the Care and Use of Laboratory Animals (NIH, Bethesda, MD, USA). The mice were randomly divided into 11 groups (*n* = 7 in each group): the DMSO, ASI (200 mg/kg), normal, DSS, low dose ASI (LASI)‐DSS, high dose ASI (HASI)‐DSS, DMSO‐DSS, DSS + ASI + OE‐Vector, DSS + ASI + OE‐WDR5, DSS + ASI + OE‐WDR5 + sh‐NC group, and DSS + ASI + OE‐WDR5 + sh‐ENO1 groups.

During Days 1–7, mice in the normal, DMSO, and ASI groups were given distilled water for 7 consecutive days, and UC was induced by replacing distilled water with 5% DSS (60316EG25, Yeasen Biotechnology Co. Ltd., Shanghai, China) for 7 consecutive days. During Days 8–14, low (100 mg/kg) or high (200 mg/kg) doses of ASI (53037ES10, Yeasen) [16] and negative control DMSO (100 mg/kg) were administered orally for the next 7 consecutive days [17]. For mice in the DSS + ASI + OE‐Vector, DSS + ASI + OE‐WDR5, DSS + ASI + OE‐WDR5 + sh‐NC, and DSS + ASI + OE‐WDR5 + sh‐ENO1 groups, 50 μL of lentivirus (1 × 10^8^ TU) was administered three times by intracolonic injection (on Days 0, 2, and 4 of DSS modeling) to achieve the gene intervention.

### 2.2 Evaluation of the Disease Activity Index (DAI) and Colon Collection

Disease severity was recorded daily and DAI was recorded using three parameters to evaluate colitis in mice: weight loss (0–4 points, 0%–20% loss), fecal consistency (0 points for normal, 2 points for loose stools, 4 points for diarrhea), and occult blood (0 points for normal, 2 points for occult blood positivity, 4 points for overt bleeding). DAI was the mean value of the above parameters. On Day 15, all mice were euthanized by intraperitoneal injection of 150 mg/kg sodium pentobarbital. The colon was resected and washed with PBS solution, and the length of the resected colon was measured, followed by photographing. For mice in the DMSO and ASI groups, liver, kidney, and heart tissues were isolated separately.

### 2.3 Pathological Assessment

After the euthanasia of mice, colon, liver, kidney, and heart samples were fixed in 4% formalin for 24 h, embedded in paraffin, cut into sections at 4‐μm, and stained with hematoxylin and eosin (C0105S, Beyotime Biotechnology Co. Ltd., Shanghai, China). Colon injury scores were calculated based on mucosal thickening (0–4), inflammatory cell infiltration (0–4), goblet cell depletion (0–4), structural disruption (0–4), and crypt loss (0–4), with a maximum score of 20 [18].

### 2.4 Cell Culture and Treatment

Normal human colon epithelial cells, NCM460 (CL0393, Fenghuishengwu, Changsha, Hunan, China), were cultured in Eagle's minimum essential medium (30–2003, American Type Culture Collection, Manassas, VA, USA) at 37°C with 5% CO_2_. OE‐WDR5 and sh‐ENO1 lentiviruses were obtained from VectorBuilder (Guangzhou, Guangdong, China). NCM460 cells were cultured in Petri dishes, infected with OE‐WDR5 alone or with sh‐ENO1, and further screened using puromycin to obtain stably infected cells. For DSS stimulation, NCM460 cells were incubated with 2.0% DSS for 24 h [19]. NCM460 cells requiring ASI treatment were cultured with 50 μg/mL ASI for 20 h.

### 2.5 Inflammatory Cytokine Analysis

After lysis of the colon tissue using RIPA buffer solution containing 1% PMSF, the supernatant was collected by centrifugation. Supernatant from the NCM460 cell culture was collected as well. The ELISA kits for mouse tumor necrosis factor‐α (TNF‐α, GEM0004‐48T, Servicebio, Wuhan, Hubei, China), interleukin (IL)‐1β (GEM0002‐48T, Servicebio), IL‐6 (GEM0001‐48T, Servicebio), and IL‐10 (E‐HSEL‐M0004, Elabscience Biotechnology Co. Ltd., Wuhan, Hubei, China) were used to examine cytokine levels in mouse samples. The ELISA kits for human IL‐6 (E‐HSEL‐H0003, Elabscience), TNF‐α (GEH0004‐48T, Servicebio), IL‐1β (E‐HSEL‐H0001, Elabscience), and IL‐10 (GEH0003‐48T, Servicebio) were used to examine cytokine levels in NCM460 cell culture supernatant. The absorbance value at 450 nm was detected using a microplate reader. The concentrations of cytokines were obtained according to the standard curves.

### 2.6 Evaluation of Oxidative Stress

Colonic tissues of DSS mice were homogenized into tissue homogenate with saline and centrifuged to harvest the supernatant for the following assay. The myeloperoxidase (MPO) activity was determined by using MPO assay kits (A044‐1‐1, Nanjing JianCheng Bioengineering Institute, Nanjing, Jiangsu, China). The samples, reagents 2 and 3, were added in a water bath at 37°C for 15 min, and reagent 4 and color developer were added for another 30‐min bath at 37°C. After a final 10‐min bath with reagent 7 at 60°C the absorbance at 460 nm was measured using a UV spectrophotometer.

The nitric oxide (NO) content was calculated using the NO assay kit as well (A012‐1‐2, JianCheng). The sample was cultured with a mixture of reagents 1 and 2 in a 37°C water bath for 60 min, treated with reagents 3 and 4 at room temperature for 40 min, and centrifuged for 10 min. The supernatant was reacted with the color developer for 10 min, and the absorbance of the resulting mixture was measured at 550 nm using a UV spectrophotometer.

### 2.7 Western Blot Analysis

Total proteins were extracted from mouse colon tissues and NCM460 cells using RIPA buffer containing 1% PMSF, and the total proteins were determined by the BCA protein assay kit (P0012, Beyotime). The protein samples were denatured with SDS gel loading buffer (AM8546G, Thermo Fisher Scientific Inc., Waltham, MA, USA) for 10 min and electrophoresed on 10% SDS‐PAGE. After electrophoresis, the separated proteins were transferred to a polyvinylidene difluoride membrane and sealed in 5% goat serum for 2 h. After being washed with TBST (60145ES76, Yeasen), the membrane was probed with the following primary antibodies at 4°C overnight, including ZO‐1 (1:1000, FNab09751, FineTest, Wuhan, Hubei, China), claudin‐3 (1:1000, ab214487, Abcam, Cambridge, MA, USA), Cleaved‐Caspase‐3 (1:1000, MBS9410752, MyBioSource Inc., San Diego, CA, USA), WDR5 (1:1000, ab307664, Abcam), H3K4me3 (1:1000, MBS8527387, MyBioSource), ENO1 (1:1000, PA5‐21387, Thermo Fisher), and β‐actin (1:1000, ab8227, Abcam). After washes with TBST buffer, the membranes were incubated with the secondary antibody, goat‐anti‐rabbit IgG (HRP) (1:2000, ab6721, Abcam) for 1 h at room temperature. Subsequently, immunoreactive bands were developed using an enhanced chemiluminescence kit (SQ101, Epizyme, Shanghai, China) and visualized.

### 2.8 Cell Counting Kit‐8 (CCK‐8)

Cell viability was detected by CCK‐8 kits (40203ES60, Yeasen). NCM460 cells were seeded in 96‐well plates. After 48 h of culture in a 5% CO_2_ incubator and different treatments, the cells were treated with 10 μL of CCK‐8 solution for 2 h. Absorbance at 450 nm was read using a microplate reader.

### 2.9 RT‐qPCR

Total RNA was extracted from NCM460 cells using TRIzol reagent (R0016, Beyotime), and DNase I was used to break down the DNA mixed with the genome. Finally, the total RNA was purified by phenol/chloroform extraction and ethanol precipitation and reverse transcribed to cDNA using the PrimeScript RT reagent Kit (RR037Q, Takara Biotechnology Ltd., Dalian, Liaoning, China). The cDNA samples were used for real‐time PCR with TB Green Premix Ex Taq II (Tli RNaseH Plus, RR820Q, Takara) on the StepOnePlus Real‐Time Fluorescence PCR System (4,376,600, Thermo Fisher). The expression levels of the target genes were normalized to the housekeeping gene GAPDH using the 2^−ΔΔCt^ method. Primers used in qPCR are listed in Table 1.

**TABLE 1:** Primer sequence for quantitative real‐time PCR.

Gene	Forward sequence (5′–3′)	Reverse sequence (5′–3′)
WDR5 (human)	AGTGCCTCAAGACTTTGCCAGC	CGATGAGCGTCTTCAGGCACTG
ENO1 (human)	AGTCAACCAGATTGGCTCCGTG	CACAACCAGGTCAGCGATGAAG
GAPDH (human)	GTCTCCTCTGACTTCAACAGCG	ACCACCCTGTTGCTGTAGCCAA
WDR5 (mouse)	CTCCTTGTGTCTGCCTCTGATG	CCTGAGACGATGAGGTTGGACT
ENO1 (mouse)	TACCGCCACATTGCTGACTTGG	GCTTGTTGCCAGCATGAGAACC
GAPDH (mouse)	CATCACTGCCACCCAGAAGACTG	ATGCCAGTGAGCTTCCCGTTCAG

Abbreviations: ENO1, enolase 1; GAPDH, glyceraldehyde‐3‐phosphate dehydrogenase; WDR5, WD repeat‐containing protein 5.

### 2.10 Chromatin Immunoprecipitation (ChIP)

The occupancy of H3K4me3 in the promoter of ENO1 was detected using a ChIP assay kit (P2078, Beyotime). OE‐WDR5‐infected NCM460 cells were fixed with 1% formaldehyde at 37°C for 10 min for cross‐linking, and after quenching the reaction by the addition of glycine, the cells were rinsed with PBS containing 1 mM PMSF. The precipitate was resuspended with SDS lysis buffer containing 1 mM PMSF, ice‐bathed for 10 min, and sonicated. The sonicated samples were centrifuged at 4°C for 5 min, and the supernatant was added to ChIP buffer containing 1 mM PMSF. After mixing with Protein A + G beads for 30 min at 4°C and centrifugation at 1000 g at 4°C, the supernatant was immunoprecipitated with antibodies against H3K4me3 (1:100, MBS8527387, MyBioSource) and WDR5 (1:50, 13,105, Cell Signaling Technologies, Beverly, MA, USA) at 4°C overnight. Protein A+G beads were added and incubated for 60 min at 4°C with oscillations. After separation of the beads, the immunoprecipitate was washed with low‐salt immunocomplex wash, high‐salt immunocomplex wash buffer, LiCl immunocomplex wash buffer, and Tris‐EDTA (TE) buffer. The solution was centrifuged at 4°C for 1 min. The supernatant was added to freshly prepared elution buffer and eluted successively for 5 min. The solution was centrifuged for 1 min to obtain a supernatant. Cross‐linking was reversed by heating at 65°C for 4 h. Finally, the samples were subjected to DNA purification. The DNA precipitates were resuspended using 40 μL TE and amplified by PCR using promoter‐specific primers for ENO1.

### 2.11 Immunohistochemistry

Paraffin‐embedded sections were deparaffinized, placed in citrate antigen retrieval buffer (PH = 6), and treated with 3% hydrogen peroxide solution to block endogenous peroxidase. After blocking with PBS containing 5% goat serum, the sections were incubated with primary antibodies to WDR5 (1:100, ab307664, Abcam) and ENO1 (1:100, PA5‐21387, Thermo Fisher) overnight at 4°C and with the secondary antibody goat anti‐rabbit IgG (1:1000, ab6721, Abcam) for 2 h. After being stained with DAB and hematoxylin, the sections were observed.

### 2.12 TUNEL Assay

Apoptosis levels were detected using the one‐step TUNEL apoptosis assay kit (C1086, Beyotime). The cells were fixed with immunol staining fix solution (P0098‐100 mL, Beyotime) for 30 min, rinsed with PBS, and incubated with enhanced immunostaining permeabilization solution (P0097‐100 mL, Beyotime) for 5 min at room temperature and with TUNEL detection solution at 37°C in the dark for 60 min. Finally, the cells were sealed with an anti‐fluorescence quenching sealing solution and then observed under the microscope. The percentage of apoptotic cells (%) to the total number of cells was counted in five random fields of view.

### 2.13 Statistical Analysis

Statistical analysis was performed using GraphPad Prism software 10.4.2 (GraphPad, San Diego, CA, USA) and is presented as mean ± standard error of the mean (SEM) of results from at least three independent experiments. The data were analyzed using an unpaired *t*‐test between two groups and a one‐way/two‐way analysis of variance, followed by Tukey's or Šídák's post hoc tests when groups were more than two. A statistical difference was considered significant at the values of *p* < 0.05.

## 3 Results

### 3.1 ASI Relieves DSS‐Induced UC in Mice

**FIGURE 1:**
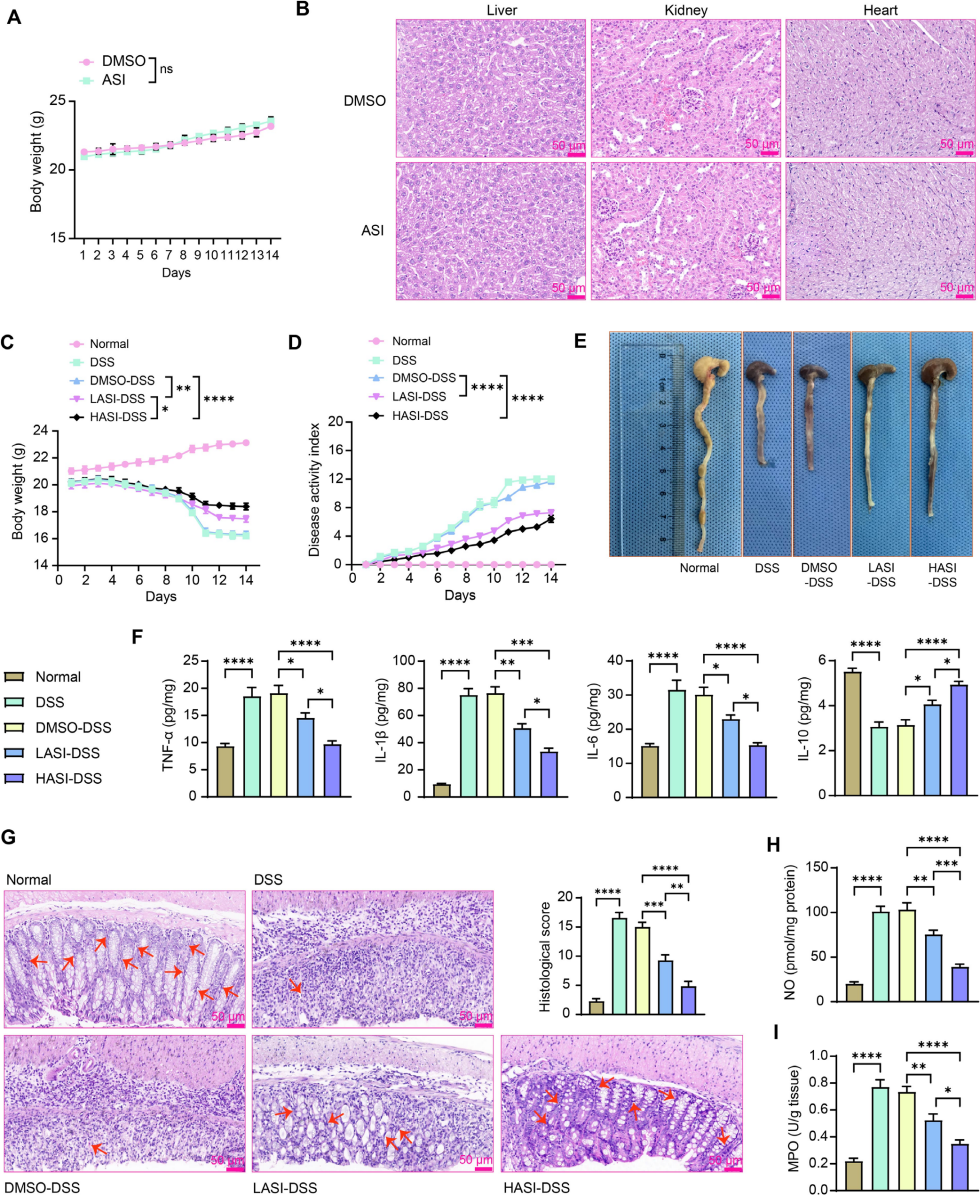
ASI alleviates UC‐like symptoms in mice induced with DSS. The body weight (A) and disease activity index (B) of normal mice treated with DMSO or ASI (200 mg/kg) were recorded during the 14‐days period. The body weight (C) and disease activity index (D) of DSS‐induced mice treated with DMSO, low dose of ASI (LASI), and high dose of ASI (HASI) were recorded during the 14‐days period. (E) Measurement of the length of the colon in mice at d 15 of modeling. (F) TNF‐α, IL‐1β, IL‐6, and IL‐10 contents in the colonic tissues of mice were examined using ELISA. (G) Analysis of colonic tissue pathology and tissue damage scores using HE staining. Detection of NO (H) and MPO content (I) in the colonic tissues of mice. The data are expressed as the means ± SEM, *n* = 7. ANOVA was applied for each comparison. *p < 0.05, **p < 0.01, ***p < 0.001, ****p < 0.0001.

First, we assessed the biotoxicity of ASI in mice by treating normal mice with high doses of ASI and evaluating the change in body weight of the mice. ASI, even at 200 mg/kg, did not have any adverse effects on the body weight of mice (Figure 1A). Further isolation of mouse liver, kidney, and heart tissues and HE staining showed that ASI did not cause damage to the organs of mice (Figure 1B), indicating that ASI possesses good biological safety. We constructed a DSS model for mice to simulate UC injury in vivo. Measuring the body weight of the mice every day revealed that compared with the body weight changes of the mice in the normal group, the mice in the DSS and DMSO‐DSS groups lost significantly more weight, and the mice in the LASI‐DSS and HASI‐DSS groups had relatively less weight loss (Figure 1C). DAI was much higher in the DSS and DMSO‐DSS groups than in the normal group, which was partially reduced by LASI and HASI (Figure 1D). After euthanasia, the length of the colon of the mice was measured. The mice in the DSS and DMSO‐DSS groups showed severe colon shortening, whereas the LASI‐DSS and HASI‐DSS groups showed less shortening of the colon (Figure 1E). As revealed by ELISA, DSS modeling contributed to a remarkable increase in pro‐inflammatory cytokines and a decrease in anti‐inflammatory factor IL‐10 in the colonic tissues of mice, while the inflammatory response was partially relieved by ASI treatments (Figure 1F). HE staining was conducted to observe the colonic tissues of mice. The histological damage was heavier in the DSS‐induced mice, and the histological damage scores of ASI‐treated mice were significantly lower (Figure 1G). The results of the NO (Figure 1H) and MPO activity (Figure 1I) assays showed that ASI was able to significantly inhibit DSS‐induced NO and MPO production. Moreover, by comparing the efficacy of ASI at low (100 mg/kg) or high (200 mg/kg) doses, we found that the therapeutic efficacy of HASI was superior.

### 3.2 ASI Alleviates DSS‐Induced NCM460 Cell Apoptosis

**FIGURE 2:**
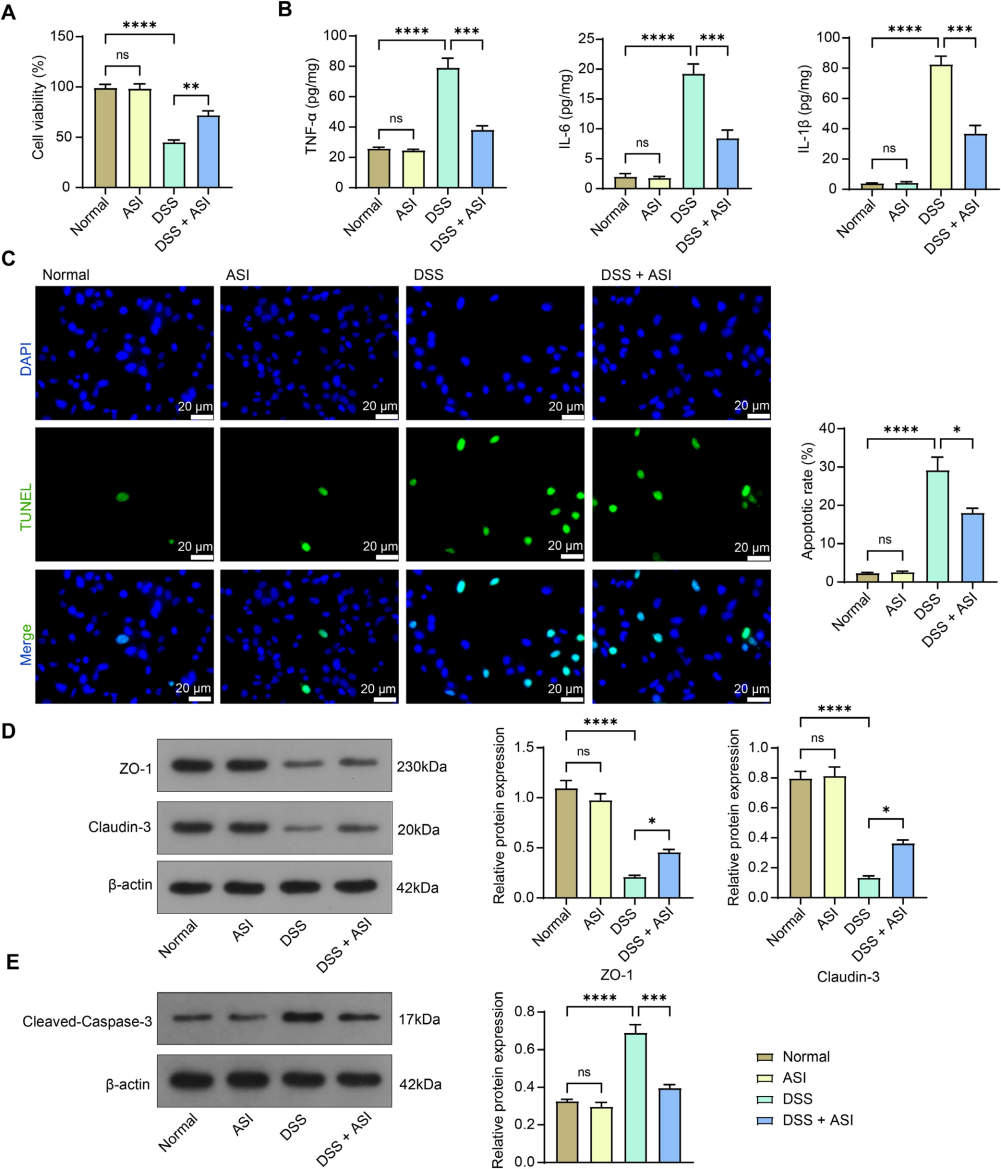
NCM460 cell damage induced by DSS is relieved by ASI. (A) The viability of NCM460 cells was examined using the CCK‐8 assay. (B) TNF‐α, IL‐6, and IL‐1β contents in NCM460 cell culture supernatant were examined using ELISA. (C) The apoptosis of NCM460 cells was examined using the TUNEL assay. (D) The protein expression of ZO‐1 and claudin‐3 in NCM460 cells was examined using Western blot assays. (E) The protein expression of Cleaved‐Caspase‐3 in NCM460 cells was examined using Western blot assays. The data are expressed as the means ± SEM, *n* = 3. ANOVA was applied for each comparison. *p < 0.05, **p < 0.01, ***p < 0.001, ****p < 0.0001.

We treated NCM460 cells with DSS to mimic the development of UC in vitro and assessed the cell viability using the CCK‐8 assay. The cell viability of DSS‐stimulated NCM460 cells was markedly reduced, and the cell viability of ASI‐treated NCM460 cells was boosted (Figure 2A). ELISA showed that TNF‐α, IL‐1β, and IL‐6 levels were significantly increased in cell culture supernatant collected from DSS‐induced cells, but ASI significantly reduced the overproduction of pro‐inflammatory cytokines (Figure 2B). The apoptosis level of the cells was detected using TUNEL assays, and the results proved that the apoptosis level of the cells induced by DSS was significantly decreased following ASI treatment (Figure 2C). Western blot assay for the expression of tight junction‐associated proteins ZO‐1 and claudin‐3 in cells showed that DSS resulted in a significant decrease in the expression of ZO‐1 and claudin‐3, which was alleviated using ASI (Figure 2D). Finally, the assessment of Cleaved‐Caspase‐3 protein expression in the cells mirrored the TUNEL assay results, where ASI repressed the protein expression of pro‐apoptosis marker Cleaved‐Caspase‐3 (Figure 2E). Meanwhile, we found that the ASI treatment had no obvious cytotoxicity to the normal NCM460 cells.

### 3.3 WDR5, a Possible Target of ASI, is Significantly Upregulated in UC Modeling In Vivo and In Vitro

**FIGURE 3:**
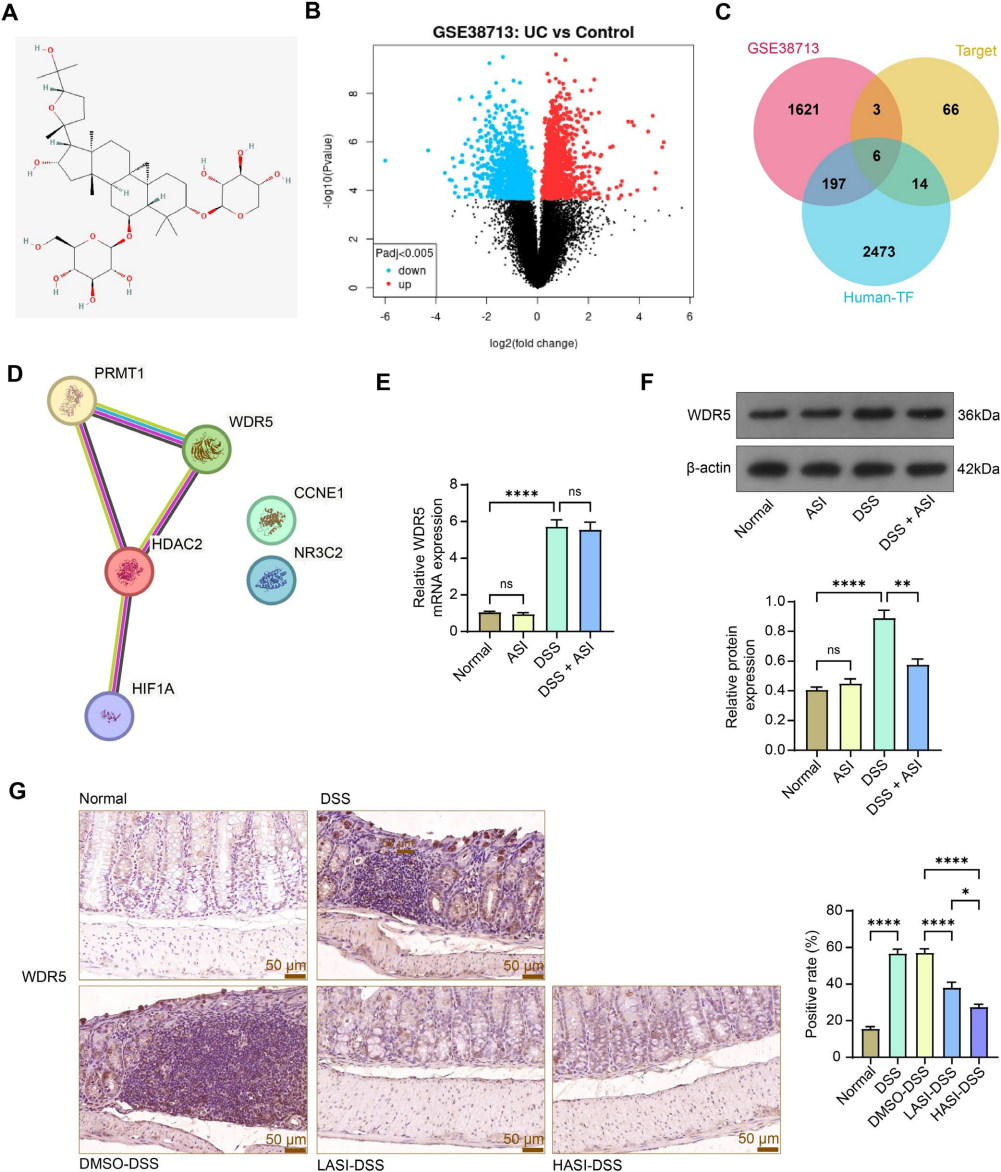
WDR5, a target of ASI, is overexpressed in UC models. (A) The chemical structural formula of ASI was obtained from PubChem Substance. (B) Transcriptional differences between colonic biopsies from patients with UC and non‐inflammatory controls in the GSE38713 dataset. (C) The intersection of differentially expressed genes in the GSE38713 dataset, targets of ASI, and human TF and TF cofactors. (D) Six intersecting interactions were subjected to protein–protein interaction plotting (connections between two protein nodes represent evidence of interactions, and more connecting lines between the two indicate a closer relationship between the predicted interactions). (E) mRNA expression of WDR5 in NCM460 cells treated with ASI or not was examined using RT‐qPCR. (F) The protein expression of WDR5 in NCM460 cells treated with ASI was examined using western blot analysis. (G) Positive rate of WDR5 in the colonic tissues of mice detected by immunohistochemistry. The data are expressed as mean ± SEM, *n* = 3 (E, F) or 7 (G). ANOVA was applied for each comparison. *p < 0.05, **p < 0.01, ****p < 0.0001.

We first obtained the chemical structural formula (Figure 3A) of ASI from PubChem Substance (https://www.ncbi.nlm.nih.gov/pcsubstance/?term=) and plotted it into Super‐PRED (https://prediction.charite.de/index.php) for target protein prediction. Thereafter, we also searched for the GSE38713 dataset with *p* adj. < 0.005 as a filter for analyzing transcriptomic differences between the colonic mucosa of 30 UC patients and 13 non‐inflammatory controls (Figure 3B). We downloaded the human transcription factors (TF) and TF cofactors in the Human TFDB (http://bioinfo.life.hust.edu.cn/HumanTFDB#!/) database. In Jvenn (https://jvenn.toulouse.inrae.fr/app/example.html), downstream targets of ASI with over 50% probability, differentially expressed genes in the GSE38713 dataset, and human TF and TF cofactors were subjected to intersection acquisition (Figure 3C). The six intersections obtained were then analyzed on the STRING (https://string‐db.org/) website. PRMT1 and WDR5 were most prominent in these protein interactions (with the most connecting lines) (Figure 3D). Since PRMT1 [20] has been reported in UC, we chose WDR5 as a downstream target of ASI for further analysis. WDR5 expression was significantly upregulated in the GSE38713 dataset (Log_2_FC = 0.494). To this point, we hypothesized that ASI mitigated UC progression by inhibiting the expression of WDR5.

To verify the prediction results, we first examined the expression of WDR5 in NCM460 cells using both RT‐qPCR and western blot analysis. As expected, the upregulation of WDR5 protein by DSS was reduced by ASI treatment, and ASI treatment under normal conditions did not significantly alter its expression (Figure 3E,F). Upon immunohistochemistry, the protein expression of WDR5 was upregulated in the colon tissues of mice induced by DSS and significantly downregulated by both LASI and HASI (Figure 3G).

### 3.4 Overexpression of WDR5 Enhances Apoptosis of NCM460 Cells Induced by DSS

**FIGURE 4:**
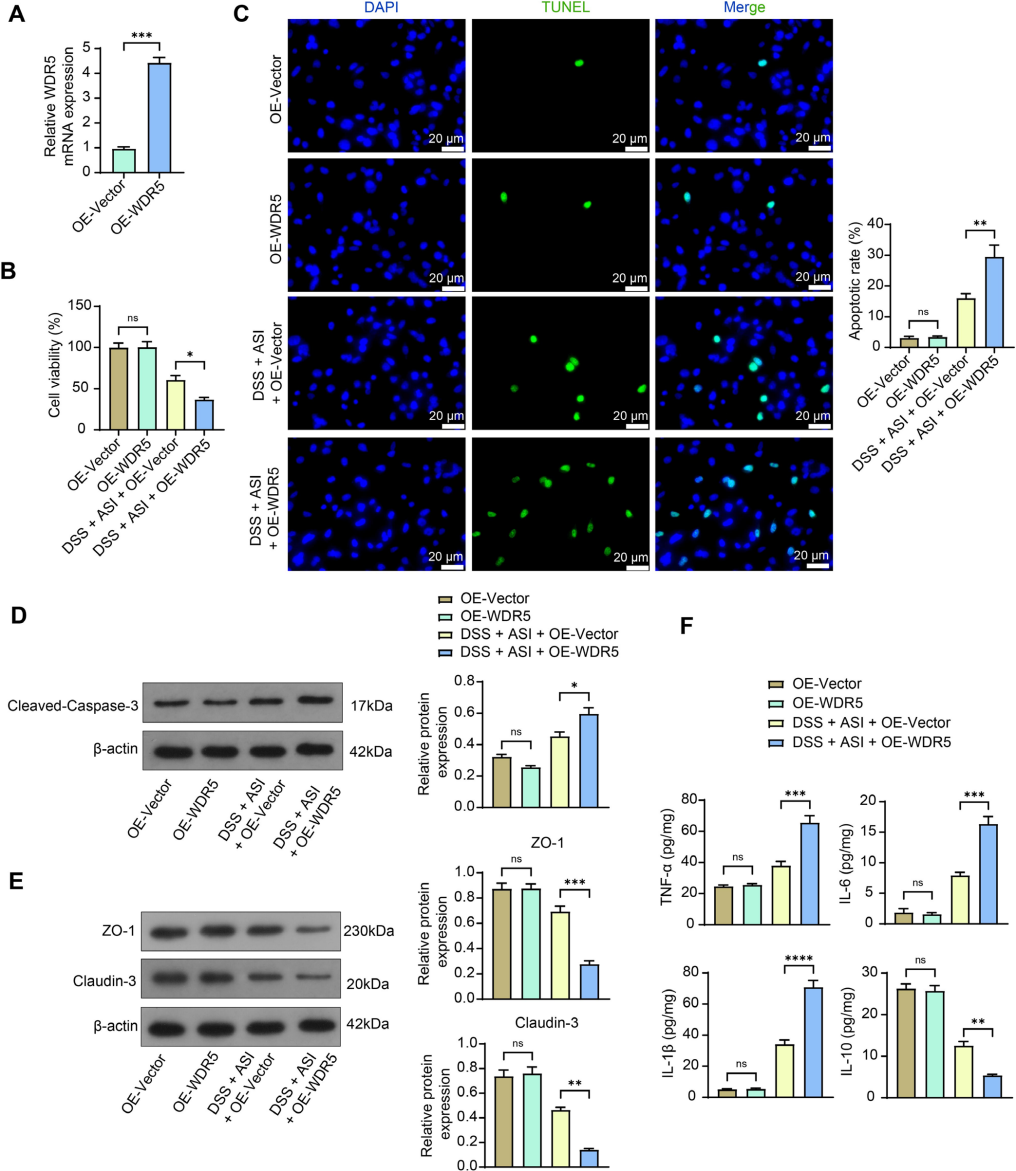
Overexpression of WDR5 weakens the effects of ASI on DSS‐induced NCM460 cells. (A) Detection of mRNA expression of WDR5 in NCM460 cells treated with ASI + OE‐Vector/OE‐WDR5 was examined using RT‐qPCR. (B) The viability of NCM460 cells was examined using the CCK‐8 assay. (C) The apoptosis of NCM460 cells was examined using the TUNEL assay. (D) The protein expression of Cleaved‐Caspase‐3 in NCM460 cells was examined using Western blot assays. (E) The protein expression of ZO‐1 and claudin‐3 in NCM460 cells was examined using Western blot assays. (F) TNF‐α, IL‐6, IL‐1β, and IL‐10 contents in NCM460 cell culture supernatant were examined using ELISA. The data are expressed as the means ± SEM, *n* = 3. An unpaired t‐test (A) or ANOVA (B–F) was applied for each comparison. *p < 0.05, **p < 0.01, ***p < 0.001, ****p < 0.0001.

NCM460 cells were infected with an overexpression lentiviral vector of WDR5 (OE‐Vector as control). First, the expression of WDR5 was significantly elevated in NCM460 cells by OE‐WDR5 infection (Figure 4A). The results of CCK‐8 and TUNEL assays showed that the cell viability was reduced considerably and apoptosis was enhanced following WDR5 overexpression after exposure to DSS and ASI (Figure 4B,C). However, the WDR5 overexpression had no impact on normal cell viability or apoptosis. Likewise, the protein expression of Cleaved‐Caspase‐3 was enhanced (Figure 4D), while the protein expression of ZO‐1 and claudin‐3 was reduced (Figure 4E) in response to the WDR5 overexpression in the cells treated with DSS and ASI. After overexpression of WDR5, the cell supernatant contained significantly higher levels of the pro‐inflammatory cytokines TNF‐α, IL‐1β, and IL‐6, and significantly lower levels of the anti‐inflammatory IL‐10 (Figure 4F). WDR5 overexpression alone did not induce tight junction protein alteration or inflammatory damage.

### 3.5 H3K4me3 Modification Catalyzed by WDR5 Increases ENO1 Expression

**FIGURE 5:**
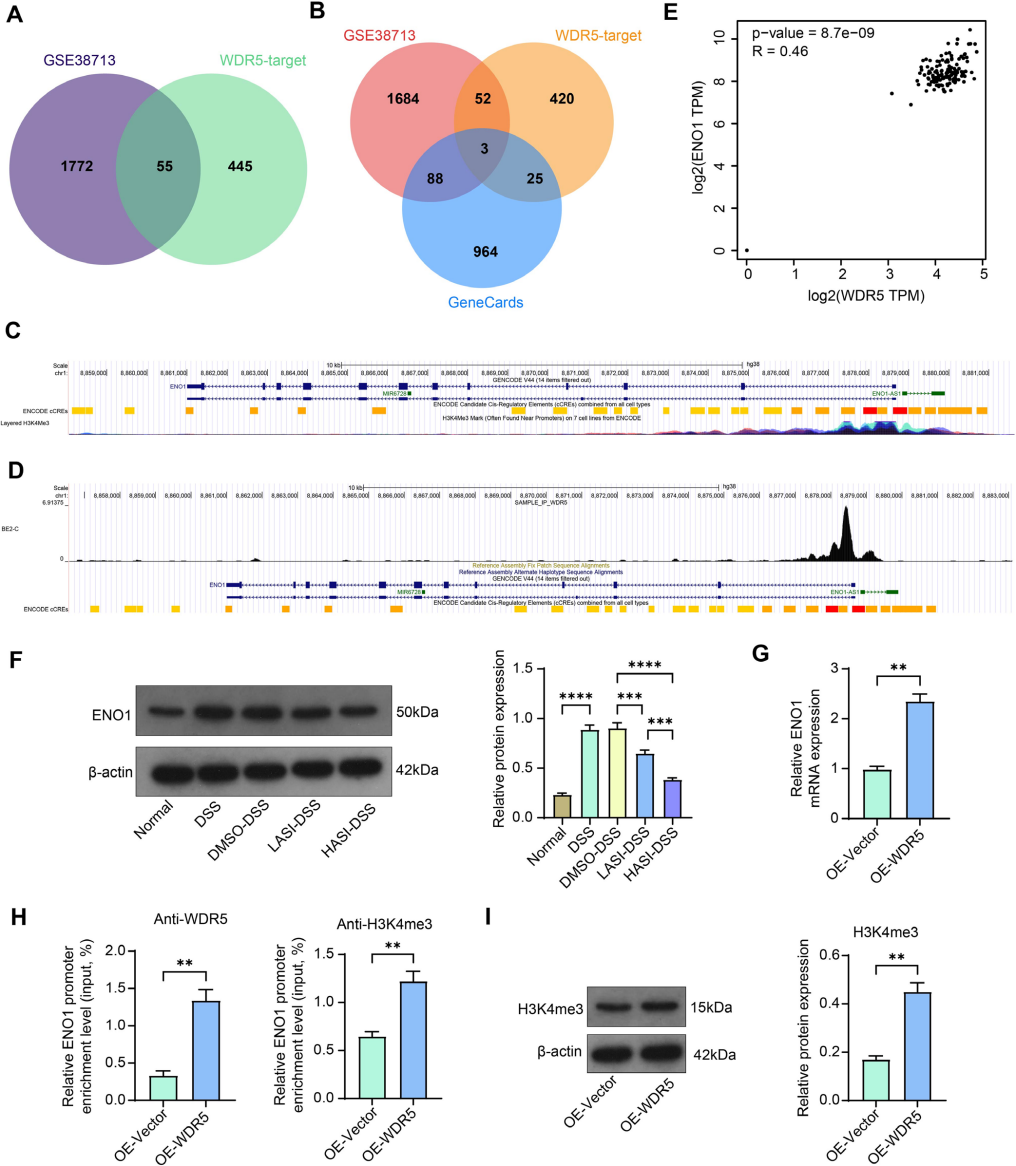
WDR5 promotes ENO1 transcription through H3K4me3 modification. (A) The intersection of differentially expressed genes in the GSE38713 dataset and WDR5 targets in the hTFtarget website. (B) The intersection of differentially expressed genes in the GSE38713 dataset, WDR5 targets in the hTFtarget website, and UC‐related genes downloaded from the GeneCards dataset. (C) ChIP‐seq analysis of H3K4me3 binding peaks on the ENO1 promoter region. (D) ChIP‐seq analysis of WDR5 binding peaks on the ENO1 promoter region. (E) Correlation analysis of WDR5 and ENO1 expression in the colon in the GEPIA dataset. (F) The protein expression of ENO1 in the colonic tissues of mice was detected by western blot analysis. (G) ENO1 mRNA expression in DSS‐stimulated NCM460 cells infected with a lentiviral vector overexpressing WDR5 was assessed using RT‐qPCR. (H) Enrichment of the ENO1 promoter region by anti‐WDR5 and anti‐H3K4me3 in NCM460 cells overexpressing WDR5 was analyzed using a ChIP assay. (I) H3K4me3 protein expression in DSS‐stimulated NCM460 cells infected with a lentiviral vector overexpressing WDR5 was examined using Western blot assay. The data are expressed as the means ± SEM, *n* = 3 (G–I) or 7 (F). An unpaired *t*‐test (G–I) or ANOVA (F) was applied for each comparison. **p < 0.01, ***p < 0.001, ****p < 0.0001.

To further explore the downstream molecular mechanisms of WDR5 in UC, we downloaded the top 500 targets of WDR5 from hTFtarget (https://guolab.wchscu.cn/hTFtarget/#!/) and intersected them with differentially expressed genes (*p* adjusted < 0.005) in the GSE38713 dataset (Figure 5A). There are a total of 55 intersection targets. To further narrow down the screening, UC‐related genes from GeneCards (https://www.genecards.org/) (relevance score top 20%) were included to obtain three intersections: CREBBP (Log_2_FC = −0.544), FGFR3 (Log_2_FC = −0.463), and ENO1 (Log_2_FC = 0.622) (Figure 5B). WDR5 interacts and recognizes the methylated fourth lysine of histone H3, histone 3 lysine 4 (H3K4), a well‐known gene activator marker [12, 13]. Therefore, significantly upregulated ENO1 in the GSE38713 dataset was selected for further analysis. Interestingly, we found by ChIP‐seq analysis of UCSC (https://genome.ucsc.edu/cgi‐bin/hgGateway) that there was indeed a significant H3K4me3 modification peak in the promoter region of ENO1 (Figure 5C), and WDR5 also had a significant binding peak (Figure 5D). In addition, WDR5 was found to be significantly positively correlated with ENO1 expression in the colon by GEPIA (http://gepia.cancer‐pku.cn/index.html) correlation analysis (Figure 5E).

Western blot experiments to detect the expression of ENO1 in the colonic tissues of mice showed that the expression of ENO1 was significantly upregulated in mice induced with DSS and downregulated considerably in mice in the LASI‐DSS and HASI‐DSS groups (Figure 5F). RT‐qPCR validation revealed that the expression of ENO1 was upregulated in NCM460 cells infected with OE‐WDR5 (Figure 5G). Using ChIP‐qPCR experiments, we confirmed that overexpression of WDR5 significantly enhanced the occupancy of WDR5 and H3K4m3 in the ENO1 promoter region (Figure 5H). Western blot experiments verified that the H3K4me3 protein expression was also increased in NCM460 cells overexpressing WDR5 (Figure 5I).

### 3.6 Silencing of ENO1 Alleviates NCM460 Cell Apoptosis and Inflammatory Response

**FIGURE 6:**
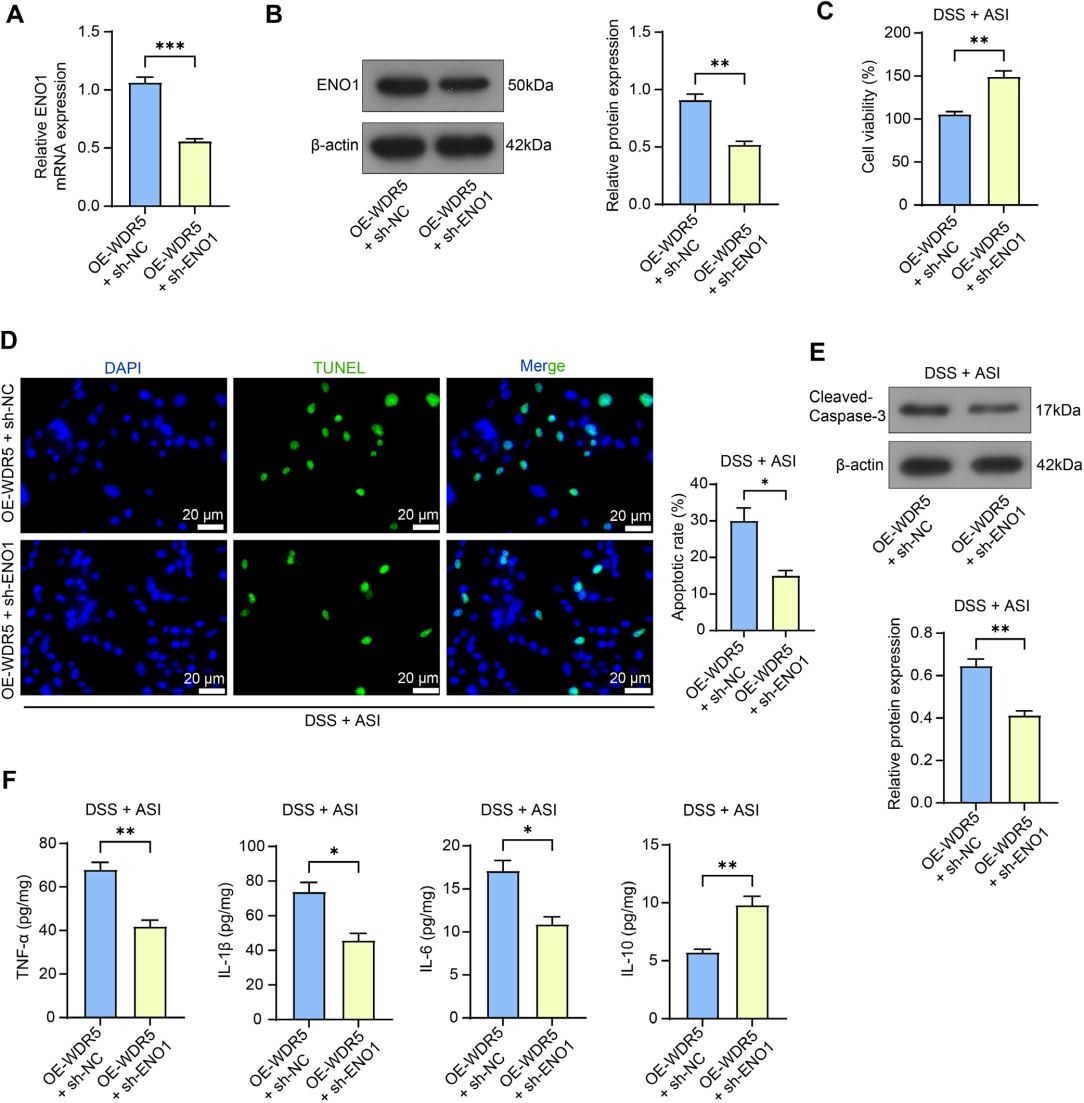
Silencing of ENO1 alleviates DSS‐induced NCM460 cell injury in the presence of WDR5 overexpression. The mRNA and protein expression of ENO1 in NCM460 cells infected with OE‐WDR5 + sh‐NC/sh‐ENO1 was examined using RT‐qPCR (A) and western blot analysis (B). (C) The viability of NCM460 cells was examined using the CCK‐8 assay. (D) The apoptosis of NCM460 cells was examined using the TUNEL assay. (E) The protein expression of Cleaved‐Caspase‐3 in NCM460 cells was examined using Western blot assays. (F) TNF‐α, IL‐6, IL‐1β, and IL‐10 contents in NCM460 cells were examined using ELISA. The data are expressed as the means ± SEM, *n* = 3. An unpaired *t*‐test was applied for each comparison. *p < 0.05, **p < 0.01, ***p < 0.001.

NCM460 cells were infected with OE‐WDR5 + sh‐NC or sh‐ENO1. It was revealed by both RT‐qPCR and western blot analysis that the downregulation of ENO1 was successful (Figure 6A,B). After DSS induction and ASI treatment, we found that the cell viability was enhanced, while the apoptosis was reduced following the downregulation of ENO1 (Figure 6C,D). Furthermore, the protein expression of Cleaved‐Caspase‐3 was also decreased (Figure 6E), which occurred concomitantly with repressed TNF‐α, IL‐1β, and IL‐6 and restored IL‐10 levels in the NCM460 cell culture supernatant (Figure 6F).

### 3.7 ASI Alleviates UC in Mice Through the WDR5‐ENO1 Axis

**FIGURE 7:**
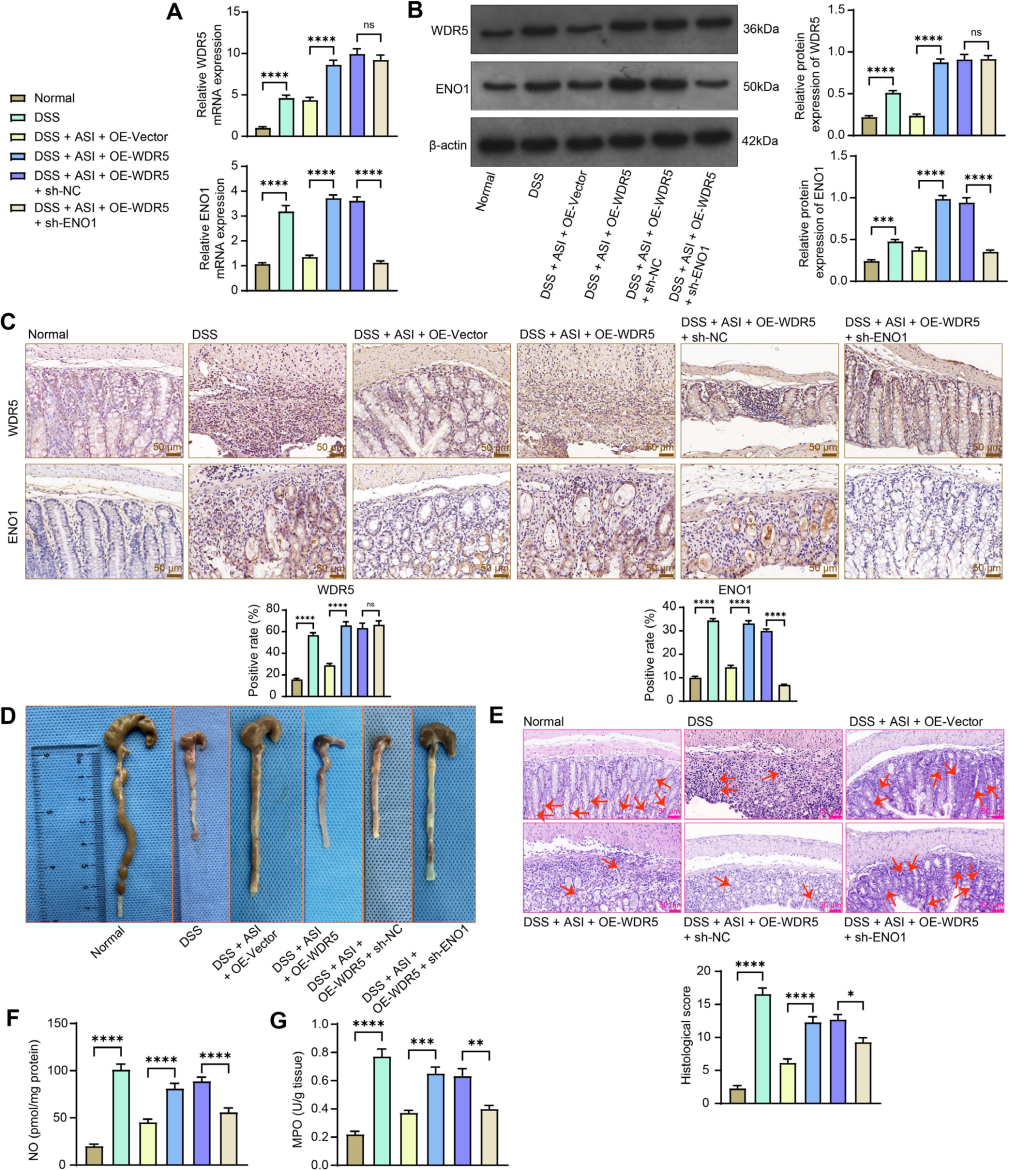
Silencing of ENO1 mitigates the UC‐like symptom in mice in the presence of WDR5 overexpression. The mRNA and protein expression of ENO1 and WDR5 in colon tissues of mice treated with OE‐WDR5 + sh‐NC/sh‐ENO1 and ASI was examined using RT‐qPCR (A) and western blot analysis (B). (C) Positive rate of ENO1 and WDR5 in the colonic tissues of mice detected by immunohistochemistry. (D) Measurement of the length of the colon in mice at d 15 of modeling. (E) Analysis of colonic tissue pathology and tissue damage scores using HE staining. Detection of NO (F) and MPO content (G) in the colonic tissues of mice. The data are expressed as the means ± SEM, *n* = 7. ANOVA was applied for each comparison. *p < 0.05, **p < 0.01, ***p < 0.001, ****p < 0.0001.

Lentiviruses were delivered into mice to upregulate the expression of WDR5 alone or simultaneously downregulate ENO1. As revealed by RT‐qPCR and western blot analysis, the ENO1 expression induced by WDR5 was decreased by sh‐ENO1, while the WDR5 expression remained (Figure 7A,B). Immunohistochemistry showed similar results, with overexpression of WDR5 inducing an increase in the positive level of WDR5 and ENO1. Moreover, a decrease in ENO1 expression was observed after knockdown of ENO1 (Figure 7C). The length of the colon of mice was measured. Severe colonic shortening was observed in the ASI + OE‐WDR5 group compared to the ASI + OE‐Vector group, and less colonic shortening was noted in the ASI + OE‐WDR5 + sh‐ENO1 group compared to the ASI + OE‐WDR5 + sh‐NC group (Figure 7D). HE staining was used to observe the colonic tissue damage of mice, and it was found that mice in the ASI + OE‐WDR5 group had severe histological damage, and the histological damage score of mice in the ASI + OE‐WDR5 + sh‐ENO1 group was significantly lower compared with that of the ASI + OE‐WDR5 + sh‐NC group (Figure 7E). NO and MPO activity measurements revealed higher NO and MPO contents in colonic tissues of mice overexpressing WDR5 and lower contents after ENO1 knockdown (Figure 7F,G). Meanwhile, overexpression of WDR5 only weakened the therapeutic effect of ASI and exacerbated the pathological symptoms of colitis in mice but did not make the therapeutic effect of ASI disappear, which may be related to the diverse targets of ASI, whose effect is not entirely dependent on the inhibition of WDR5. In addition, knockdown of ENO1 alleviated the exacerbation of disease symptoms by WDR5 overexpression.

## 4 Discussion

**FIGURE 8:**
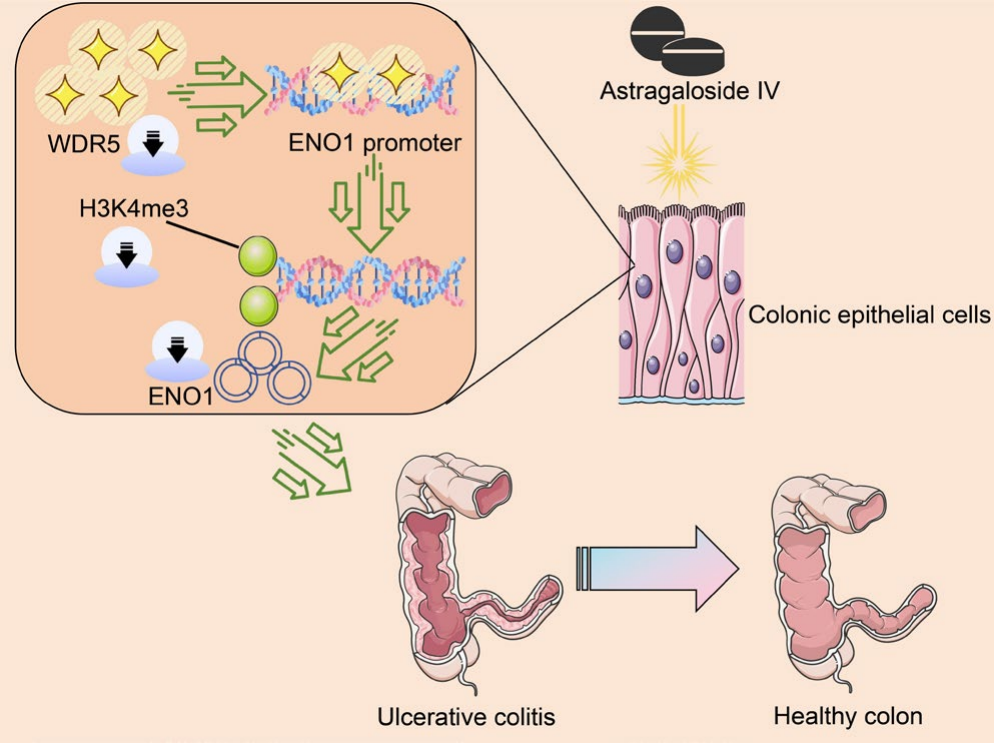
Possible mechanism of action of ASI in UC. Inhibition of WDR5‐mediated modification of H3K4me3 on the ENO1 promoter by ASI hinders transcriptional activation of ENO1 to alleviate symptoms of UC.

We demonstrated here a new epigenetic mechanism by which ASI repressed ENO1 expression by losing the enrichment of WDR5‐mediated histone methylation at the ENO1 promoter region (Figure 8).

ASI has recently been revealed to ameliorate cardiac hypertrophy in animals by rescuing hypoxia‐induced cardiac apoptosis and promoting cell viability [21]. ASI improved isoprenaline‐induced gut microbial dysbiosis [22]. ASI ameliorated indomethacin‐induced intestinal inflammation in rats by inhibiting the activation of NLRP3 inflammasome and reducing the release of IL‐1β and IL‐18 [23]. Moreover, ASI attenuated the clinical activity of DSS‐induced colitis and resulted in the phenotypic transition of macrophages from immature pro‐inflammatory macrophages to mature pro‐resolving macrophages [24]. Cytokines, including IL‐1β, IL‐6, and TNF‐α, are expressed at relatively higher levels in the intestinal tissues of patients with UC [25], and MPO‐mediated damage is associated with some disorders in people, including inflammatory bowel disease [26]. Our in vivo evidence showed that both high and low doses of ASI can effectively repress the inflammatory response and oxidative stress in the colon tissues of mice induced with DSS, in addition to the in vitro findings that ASI restored ZO‐1 and claudin‐3 protein expression. Besides that, ASI has been shown to suppress proliferation, induce tumor cell apoptosis, and reduce drug resistance in colorectal cancer [27], indicating the further role of ASI in treating UC‐induced carcinogenesis.

Even though ASI has been reported to effectively prevent and alleviate the clinical symptoms of DSS‐induced mice, including weight loss, DAI soaring, and colon length shortening, the main focus has been on the homeostasis of Th17/Treg [28]. Therefore, the downstream targets have not been identified. Here, the H3K4‐specific histone methyltransferase WDR5 was determined as a target of ASI by combining several online algorithms. WDR5 is of particular importance: its propeller‐shaped WD interaction domain interacts with a litany of proteins and some lncRNAs [29]. Consequently, WDR5 is recruited by different RNA‐binding proteins and lncRNAs to the promoter region of the downstream targets, thereby enabling H3K4me3 modification and transcriptional activation in varying inflammatory diseases, including liver fibrosis and Alzheimer's disease [30, 31]. Moreover, the administration of MM102, a selective MLL1/WDR5 complex inhibitor, attenuated tubular injury and apoptosis, while repressing WDR5 and H3K4me3 [32]. However, its functional role under pro‐inflammatory conditions has not been well described. Interestingly, Katakia et al. found that reducing H3K4me3 in endothelial cells undergoing inflammation attenuated endothelial inflammation and apoptosis [33]. We therefore anticipated that its role in UC was related to its downstream targets.

We identified ENO1 as the target of WDR5 in UC, whose expression was found to be controlled by WDR5‐mediated H3K4me3 modification. Moreover, protein arginine methyltransferase 5 symmetrically dimethylated ENO1 at arginine 9 to promote active ENO1 dimer formation [34]. However, WDR‐mediated H3K4me3 modification of ENO1 has not been revealed before. The expression of ENO1 was closely associated with many diseases, including Alzheimer's disease, diabetes, as well as cancers [35]. The overproduction of pro‐inflammatory cytokines TNF‐α, IL‐1β, and IL‐6 was observed in concanavalin A‐activated peripheral blood mononuclear cells and macrophages extracted from patients with rheumatoid arthritis induced by ENO1 [36]. In addition, cleaved‐Caspase‐3 was observed in the thyroid, the levels of IL‐6 were increased, and endothelial tight junction proteins were decreased in the brain of ENO1‐immunized mice [37]. Furthermore, hypoxic pulmonary hypertension was associated with an increased level of ENO1, and targeting ENO1 might reduce experimental hypoxic pulmonary hypertension by improving endothelial and mitochondrial dysfunction via the PI3K/AKT signaling pathway [38]. Considering that ASI has been reported to block the PI3K/AKT signaling to improve intestinal epithelial barrier in UC [10], we anticipated that ENO1, controlled by ASI‐mediated WDR5, might influence tight junction integrity through the PI3K/AKT signaling pathway.

The present study also has some limitations. One of them is that we were able to conduct the bioavailability and pharmacokinetics evaluation of ASI. Another limitation is that the other two targets of WDR5 screened out in this study, CREBBP and FGFR3, have been involved in the UC‐related carcinogenesis [39, 40]. Thus, it is also worth exploring whether these two genes might contribute to the therapeutic effects of ASI in the future.

## 5 Conclusion

Collectively, our study adds to the accumulating evidence that ASI ameliorates UC induced by DSS. Our study first reveals that ASI downregulates the expression of WDR5 and the downstream ENO1 expression, thereby alleviating the pro‐inflammatory response and oxidative stress in the colon tissues of mice. Our data raise strong evidence for the application of ASI in the treatment of UC.

## Conflicts of Interest

The authors declare no conflicts of interest.

## Data Availability

The data are available from the corresponding author for reasonable requests.
